# Reduction of active Fel d1 from cats using an antiFel d1 egg IgY antibody

**DOI:** 10.1002/iid3.244

**Published:** 2019-03-09

**Authors:** Ebenezer Satyaraj, Cari Gardner, Ivan Filipi, Kerry Cramer, Scott Sherrill

**Affiliations:** ^1^ Nestlé Purina Research St. Louis MO

**Keywords:** cat allergy, egg antibody, IgY

## Abstract

**Background:**

Fel d1 is the most important allergen from cats. Fel d1 is produced primarily in saliva and spread to the haircoat during grooming and then transferred to the environment via hair and dander.

**Objectives:**

A novel approach to reducing allergenic Fel d1 exposure was evaluated, involving binding the Fel d1 with an anti‐Fel d1 polyclonal egg IgY antibody. The hypothesis was that hair from cats who had been fed foods containing anti‐Fel d1 IgY would show a significant reduction in active Fel d1 (aFel d1).

**Methods:**

Hair collected from 105 cats completing a 12‐week study was evaluated for aFel d1 via ELISA. Hair was collected four times over a 2‐week baseline period, then weekly during the 10 week treatment period during which cats consumed a food containing the anti‐Fel d1 IgY.

**Results:**

Baseline aFel d1 (μg/g hair) varied greatly among the cats in this study. From week 3, there was a significant reduction in mean aFel d1 with an overall average decrease of 47% by week 10, ranging from a 33–71% decrease vs baseline. Cats with the highest baseline aFel d1 showed the greatest decrease in aFel d1.

**Conclusions & Clinical Implications:**

Feeding anti‐Fel d1 IgY to cats successfully reduced aFel d1 on their haircoat with the greatest decreases observed in cats with initially high levels. Feeding a diet with anti Fel d1 IgY significantly reduced the active Fel d1 on the hair of cats.

**Abbreviations**
aFel d1: active Fel d1
CV: coefficient of variation
IgE: Immunoglobulin E
SD: standard deviation

## 1 INTRODUCTION

Cats produce a number of potential allergens, with Fel d1 being the most important.^1^, ^2^ Fel d1 is a small glycoprotein, approximately 35–39 kDa in size, produced by the salivary and sebaceous glands of cats, with the highest concentrations found in saliva.^3^ As cats groom, Fel d1 is distributed within the haircoat and can then be shed with the cat hair and dander. As may be expected, there is a strong correlation between salivary concentrations of Fel d1 and the Fel d1 found on cat hair.^4^ In addition, its small size and structure allows Fel d1 to be easily and continuously airborne for long periods of time making it one of the easiest allergens to inhale.^5^, ^6^ Its molecular structure also allows it to adhere to fabrics, carpet and upholstered furniture.^6^, ^7^, ^8^, ^9^, ^10^ These characteristics can make it difficult to remove Fel d1 from homes and allows it to travel on clothing and other items from cat owning households to locations where no cat is present. Fel d1 has been found in homes and buildings without cats, especially in communities where cat owners live.^11^ Moreover, many cat allergic cat owners find the removal of a pet cat to be unacceptable.^12^

IgY is an avian immunoglobulin equivalent to mammalian IgG, and is naturally produced by chickens. These IgY antibodies are transferred and concentrated in egg yolks to provide passive immunity for offspring. As a result, large quantities of IgY antibodies accumulate in chicken eggs and can be used to deliver antigen‐specific IgY in food. The IgY attaches to active binding sites on targeted proteins, effectively reducing their antigenicity. Multiple studies have proven the safety and efficacy of oral administration of IgY including reducing diarrhea in domestic animals.^13^

Using a chimeric ELISA assay, we previously demonstrated that anti‐Fel d1 IgY dose‐dependently blocked Fel d1 when added to samples of cat saliva, and blocked IgE‐mediated degranulation in a humanized rat basophil assay.^14^ The objective of this study was to test the efficacy of the anti‐Fel d1 IgY for reducing immunologically active Fel d1 when fed to cats. The going‐in hypothesis was that hair taken from cats fed foods containing anti‐Fel d1 IgY would show a significant reduction in active Fel d1 (aFel d1) measured via ELISA binding.

## 2 METHODS

### 2.1 Study cats

The study was conducted at an external facility. The study protocol was reviewed and approved by the Institution's Animal Care and Use Committee and complied with all regulations set forth in the USDA Animal Welfare Act.^15^

One hundred and six healthy domestic shorthair cats, ranging in age from 7 months to 17 years and representing both males and females (46 neutered males, 3 intact males, 54 spayed females and 3 intact females) were recruited for the study. Cats were individually housed in accommodations that met or exceeded the requirements set forth in the Animal Welfare Act. Rooms were maintained between 50° and 85° F and were set to a 12 hr light/dark cycle. Cats were individually fed to maintain body weight, with food available up to 22 hr daily, and water was available ad libitum.

Cats were evaluated twice daily by trained personnel to ensure their good health and wellbeing, and veterinary care was provided as needed. All cats received a veterinary physical examination prior to the start and again at the conclusion of the study. Following completion of the study, all cats were returned to the facility's general cat population.

### 2.2 Experimental design

Cats were acclimated for 4 weeks prior to beginning the study. This was followed by a 2 week control period and 10 week test period. Prior to the control period and test period, the cats’ home and bedding were all washed thoroughly to reduce environmental contamination. The control diet and test diets were formulated and manufactured by Nestlé Purina PetCare Company. The formulations were created to meet or exceed the maintenance nutrient requirement based upon the guidelines of the Association of American Feed Control Officials. During the control period, cats were individually fed a complete and balanced control diet in amounts adequate to maintain body weight. During the test period, cats were fed the same diet supplemented with anti‐Fel d1 IgY. The test diet was formulated to contain 8 ppm anti‐Fel d1 IgY. Body weight was measured weekly and the amount of food provided was adjusted as needed to maintain ideal body weight.

### 2.3 Sample collection and Fel d1 analysis

Hair was collected for Fel d1 evaluation by brushing each cat predominantly on their front, shoulders and sides, areas where cats groom most frequently. A new brush was used for each cat and for each collection. Hair collections were performed twice weekly during the 2 week control period and weekly during the 10 week test period. Brushes were stored at −20°C until ready to ship for analysis. Approximately 100 mg hair was collected from each cat's brush and quantitatively analyzed for aFel d1 reactivity using an ELISA kit (6F9/3E4: Indoor Biotechnologies, Charlottesville, Virginia) by Indoor Biotechnologies, Charlottesville, VA, USA.

### 2.4 Statisitical analysis

To examine diet effects with repeated measures, including aFel d1, body weight, and food consumption, linear mixed effect models were conducted using the *lme4* package in *R*.^16^ Cat data was entered as a random effect where the intercept was allowed to vary by cat and Satterthwaite approximation of degrees of freedom was used to calculate the *P*‐values. Planned contrasts were constructed where each test week was compared to baseline. *P*‐values were adjusted using the single‐step method. Analyses were considered to be significant at *P* < 0.05. Follow up analysis was performed to determine any impact on the diet effect based on relative (baseline) aFel d1 production. For this analysis, cats were divided into quartiles based on their mean baseline aFel d1 level. Linear regression was used to estimate both the initial level (intercept) and change over time (slope) for each cat's aFel d1. The relationship between change over time and baseline levels of aFel d1 was then examined using an ANOVA with Tukey Post‐Hoc Tests.

## 3 RESULTS

One hundred five cats completed the study, with one cat having been removed due to a fractious personality. During the course of the study nine cats received medical care for minor health conditions including infectious conjunctivitis and ear problems, but common to a typical cat population. All other cats remained in good health.

Mean body weight remained stable on average throughout the study. There were some variations in body weight during the early weeks of the study but returned to baseline by the end of the study period as the amount of food offered was gradually decreased (Table 1).

**Table 1:** Means and SEs by week for body weight (kg), food consumption (g), and aFel d1 (μg/g hair)

	Body weight		Food consumption		aFel d1	
	Mean	SE	Mean	SE	Mean	SE
Baseline	3.74	0.04	65.20	0.80	222.01	12.81
Week 1	3.78*	0.06	62.46*	1.14	186.24**	25.67
Week 2	3.77*	0.06	60.18*	1.05	195.38	20.49
Week 3	3.78**	0.06	58.32*	1.15	107.59*	11.44
Week 4	3.78*	0.06	57.07*	1.09	145.12*	20.47
Week 5	3.77*	0.06	55.22*	1.09	143.80*	17.64
Week 6	3.77**	0.06	53.98*	1.09	90.79*	9.86
Week 7	3.76	0.07	52.91*	1.09	79.42*	6.91
Week 8	3.74	0.07	52.30*	1.02	133.51*	16.83
Week 9	3.75	0.07	51.64*	1.01	73.33*	12.15
Week 10	3.74	0.07	50.97*	1.05	133.38*	16.56

*Significantly different from baseline, *P* < 0.001; ** Significantly different from baseline, *P* < 0.05.

**Figure 1:**
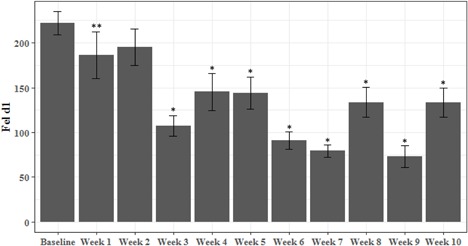
Active Feld1 (aFel d1) (μg/g hair) means and SE across weeks. Means were significantly reduced from baseline at week 1 (*P* < 0.05) and weeks 3 through 10 (*P* < 0.001).

Beginning with the first week of the test period, there was a steady decrease in aFel d1 levels (μg/g hair). The aFel d1 levels continued to decline showing significantly lower levels versus baseline from week 3 onwards through to the end of the study (*P* < 0.05) (Figure 1 and Table 1). The data analyzed on completion of the study showed an average decrease in aFel d1 of 47%, with a range between 33% and 71% from baseline. Importantly, half of the cats had at least a 50% reduction in aFel d1 and 86% had a reduction of at least 30% from baseline.

**Figure 2:**
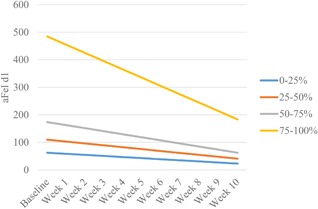
Change in aFel d1 (μg/g hair) means over time based on initial concentations, shown as simple linear regression. The slope was significantly steeper for those cats in the highest quartile (*P* < 0.001) but did not differ among the three lower quartiles (*P* > 0.1).

Baseline aFel d1 was highly variable among cats, ranging from a mean (±s.d.) of 64.4 ± 20.8 μg/g hair in the lowest quartile to 533.3 ± 330.0 μg/g hair in the highest quartile (Table 2). The decrease in aFel d1 was related to baseline levels, such that those with the highest baseline aFel d1 showed the greatest decreases (*β* = −13.37, *t* = −22.89, *R* ^2^ = 0.84, *P *< 0.001). Cats in the quartile with the highest level of aFel d1 responded with a significantly (*P* < 0.001) steeper decrease in aFel d1 over time compared with other groups (Figure 2). The slopes of the decrease in the three lower quartiles did not differ significantly (*P* > 0.10) from each other. Likewise, although the final means for all quartiles were lower from their respective baseline, the means of the three lower quartiles did not differ from each other at the end of the study (*P* > 0.73) (Table 2).

**Table 2:** Means and SD by week for aFel d1 (μg/g hair) based on baseline quartiles

	Quartile 1		Quartile 2		Quartile 3		Quartile 4	
	Mean	SD	Mean	SD	Mean	SD	Mean	SD
Baseline	64.4*	20.8	116.2*	18.9	179.2*	21.4	533.3	330.0
Week 1	57.1	39.3	98.7	47.8	160.8	110.2	433.3	426.3
Week 2	74.7	38.9	102.9	41.6	188.1	103.7	420.4	303.4
Week 3	33.3	19.0	68.3	47.8	101.3	78.4	230.3	158.3
Week 4	35.8	14.8	73.7	32.7	111.0	34.3	364.2	333.6
Week 5	41.6	23.1	77.9	36.3	130.8	54.2	328.8	282.0
Week 6	30.8	19.1	52.1	23.6	81.8	33.8	200.8	149.1
Week 7	28.7	11.1	50.6	22.5	67.8	28.8	172.6	81.0
Week 8	40.1	19.4	65.3	39.7	92.3	45.0	339.9	243.7
Week 9	18.1	9.4	31.2	10.7	51.1	30.6	195.0	205.7
Week 10	38.5*	24.0	71.6*	33.0	116.4*	96.3	310.6	249.3

Differences between quartiles were evaluated at baseline and week 10; only quartile 4 differed.

* Significantly different from Quartile 4, *P* < 0.001.

## 4 DISCUSSION

This study documented the efficacy of a novel approach for reducing the immunologically active cat allergen: aFel d1. This protein is produced in the cat's salivary and sebaceous glands with the greatest concentrations found in saliva. The cats distribute Fel d1 onto their skin and haircoat as they groom. This study confirmed that feeding cats a food containing anti‐Fel d1‐ IgY from chicken eggs was able to significantly reduce the active Fel d1 on the cats’ hair.

Fel d1 contains at least three IgE specific binding sites, and multiple IgE binding epitopes are required for allegen cross‐linking of mast cells and basophil bound IgEs and subsequent cellular degranulation. It has previously been shown that polyclonal anti‐Fel d1 IgG from rabbit sera blocked Fel d1 binding more effectively than monoclonal anti‐Fel d1 IgG, whereas the anti‐Fel d1 IgY used in this study was as effective as the polyclonal IgG at blocking Fel d1.^15^ The IgG or IgY binding of these multi‐epitope sites on Fel d1 appears to compete with IgE for binding, likely sequestering and neutralizing the allergen as has been demonstrated for human blocking antibodies.^17^ In an in vitro evaluation using a humanized rat basophil assay, this anti‐Fel d1‐ IgY blocked IgE mediated histamine release in a dose‐dependent manner.^15^

In the current study, 86% of the cats showed a reduction in aFel d1 of at least 30%, and half the cats showed a reduction of at least 50%. Perhaps more importantly, those cats that had the highest initial aFel d1 levels showed the greatest decrease. Baseline Fel d1 was highly variable among cats, indicating that some cats naturally produce less Fel d1 than others. Week to week variation was higher in cats with high Fel d1 production compared to those cats with lower Fel d1 levels, consistent with what has been observed previously.^4^ In that study, cats with naturally low salivary Fel d1 levels remained low over the course of a 1 year study, while high producers were highly variable in the levels of their salivary Fel d1, yet remained high on average. In this study, both the mean and the variation were reduced in the high producing cats, and the mean aFel d1 was reduced even in the lower Fel d1 producing cats. Based on previous data, it is unlikely that the decreases observed within this study were due to factors other than the anti‐Fel d1 IgY.

A common recommendation to remove the pet cat as the source of Fel d1 from the home will result in a gradual reduction in environmental Fel d1. However previous research has shown that some Fel d1 particles can remain in the environment for up to 5 months following the removal of cats from homes.^18^ As cats groom, Fel d1 coming from the saliva is distributed within the haircoat and can then be shed with cat hair and dander, ultimately distributing the allergen throughout the home environment. In a study evaluating effects of reducing environmental Fel d1 levels, Björnsdottir et al,^19^ showed that reducing Fel d 1 levels in the home by only 6.8% of baseline levels significantly improved symptoms of nasal allergy. Therefore, a reduction in aFel d1 from saliva can be expected to significantly reduce aFel d1 on hair and subsequently in the environment, with out the need to remove the pet cat from the home and will likely improve allergy symptoms.

## ETHICS APPROVAL AND CONSENT TO PARTICIPATE

The protocol for this study was reviewed and approved prior to study initiation by the Summit Ridge Farms’ Institutional Animal Care and Use Committee (IACUC) (USDA Registration No. 23‐R‐0126), and was in compliance with the Animal Welfare Act.

## CONFLICTS OF INTEREST

All authors are employed by Nestlé Purina Research, which funded this study in full.
